# Wide QRS‐T angles are associated with markers of increased inflammatory activity independently of hypertension and diabetes

**DOI:** 10.1111/anec.12781

**Published:** 2020-07-08

**Authors:** Mikael Sandstedt, Lennart Bergfeldt, Joakim Sandstedt, Annika Lundqvist, Emanuel Fryk, Per‐Anders Jansson, Göran Bergström, Lillemor Mattsson Hultén

**Affiliations:** ^1^ Region Västra Götaland Department of Clinical Chemistry Sahlgrenska University Hospital Gothenburg Sweden; ^2^ Department of Laboratory Medicine Institute of Biomedicine Sahlgrenska Academy University of Gothenburg Gothenburg Sweden; ^3^ Region Västra Götaland Department of Cardiology Sahlgrenska University Hospital Gothenburg Sweden; ^4^ Department of Molecular and Clinical Medicine Institute of Medicine Sahlgrenska Academy University of Gothenburg Gothenburg Sweden; ^5^ Region Västra Götaland Gothia Forum Sahlgrenska University Hospital Gothenburg Sweden

**Keywords:** cytokines, inflammation, QRS‐T angles, sudden cardiac death, vectorcardiography, white blood cells

## Abstract

**Background:**

Wide QRS‐T angles and inflammatory activity are markers of future cardiovascular events including sudden cardiac death (SCD). The association between wide QRS‐T angles and inflammatory activation is however not fully understood.

**Methods:**

1,094 study participants of both sexes, 50–64 years old, were included from a randomly selected population‐based cohort as a part of the Swedish CArdioPulmonary bioImage Study (SCAPIS) pilot study. Serum samples were analyzed for markers of inflammation, cardiac wall stress/injury, and the metabolic syndrome. Wide QRS‐T angles were defined using Frank vectorcardiography. Variables were analyzed through unsupervised principal component analysis (PCA) as well as Orthogonal Projections to Latent Structures (OPLS) modeling. In addition, a subset of study participants was analyzed in a post hoc matched group design.

**Results:**

Wide QRS‐T angles correlated positively with markers of inflammation, cardiac wall stress/injury, the metabolic syndrome, and male sex in both PCA and OPLS models. In the matched post hoc analysis, participants with wide QRS‐T angles had significantly higher counts of white blood cells (WBC) and neutrophils in comparison with matched controls. WBC as well as the number of neutrophils, monocytes, basophils, eosinophils and levels of C‐reactive protein, IL‐1, IL‐4, IL‐6, TNF‐α, and NT‐pro‐BNP were also significantly higher in comparison with healthy controls.

**Conclusions:**

Markers of inflammatory activation and cardiac injury/wall stress were significantly higher in the presence of wide QRS‐T angles. These results corroborate an association between abnormal electrophysiological function and inflammatory activation and may have implications for the prediction of SCD.

## 1 INTRODUCTION

Sudden cardiac death (SCD) is a common cause of death worldwide in patients suffering from ischemic heart disease with or without heart failure, as well as in previously asymptomatic individuals (Zheng, Croft, Giles, & Mensah, 2001). Although several mechanisms exist, electrical disturbances leading to ventricular tachycardia (VT) or fibrillation (VF) dominate (Al‐Khatib et al., 2018). When out‐of‐hospital cardiac arrest occurs, the prognosis is dismal even when cardiopulmonary resuscitation is initiated before the emergency medical service arrives (Hasselqvist‐Ax et al., 2015). Increased knowledge about mechanisms contributing to SCD is therefore necessary in order to improve risk prediction and SCD prevention.

Presently, most identified risk factors for SCD have a high‐negative predictive value, while the positive predictive value rarely exceeds 20% (Huikuri et al., 2003). Electrophysiological abnormalities reflected by the QRS‐T angles are known to predict cardiovascular events including SCD in various cohorts (Lingman et al., 2016; Oehler, Feldman, Henrikson, & Tereshchenko, 2014; Waks et al., 2016; Yamazaki, Froelicher, Myers, Chun, & Wang, 2005). Furthermore, experimental studies have demonstrated that inflammatory mechanisms may directly contribute to the development of VT/VF by influencing the electrophysiological function of the myocardium through several mechanisms (Baum, Long, Cabo, & Duffy, 2012; Fernandez‐Velasco, Ruiz‐Hurtado, Hurtado, Moro, & Delgado, 2007; Petkova‐Kirova et al., 2006).

Our general hypothesis was that a set of risk markers—including electrophysiological, structural/mechanical, and biochemical factors—may improve prediction of SCD. This study was carried out as a first step, exploring an association between abnormal electrophysiological function and inflammatory activity. The association between QRS‐T angle width, markers of inflammation, cardiac wall stress/injury, the metabolic syndrome, and a panel of inflammatory cytokines was therefore investigated in a cross‐sectional study.

## 2 METHODS

### 2.1 Study design and population

**FIGURE 1:**
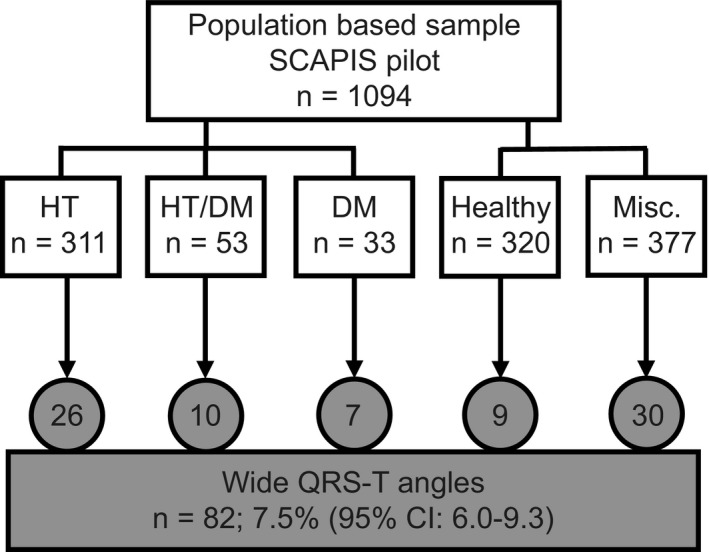
Study flow chart. DM, diabetes mellitus; HT, hypertension; Misc., miscellaneous participants not eligible for matching due to various diseases

Randomly selected citizens living in Gothenburg, Sweden, were enrolled in The Swedish CArdioPulmonary bioImage Study (SCAPIS) pilot study. Participants of both sexes, aged 50–64 years, underwent extensive standardized characterization according to a 2‐day program as described previously (Bergstrom et al., 2015). Informed consent was obtained from each participant. Out of a total of 1,111 participants, there were 1,094 with noninvasive electrocardiographic recordings according to the Frank vectorcardiography system (VCG) which were used to define the presence of wide QRS‐T angles, reflecting electrophysiological abnormality, and increased risk for SCD (Lingman et al., 2016; Oehler et al., 2014; Waks et al., 2016; Yamazaki et al., 2005; Figure 1). All data that support the findings of this study are available from the corresponding author upon reasonable request.

**FIGURE 2:**
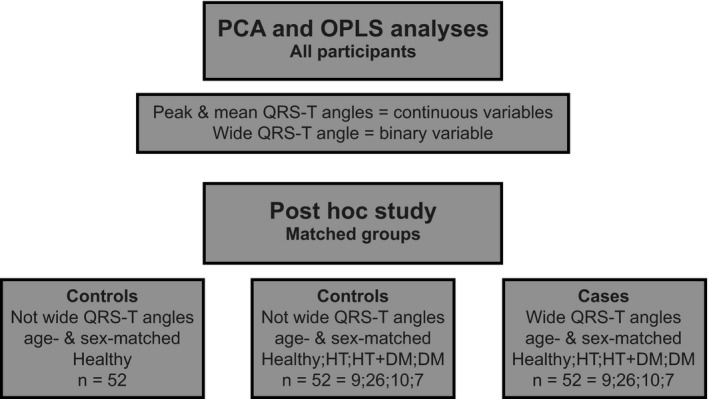
Study group overview. DM, diabetes mellitus; HT, hypertension

In the first and major analysis, all 1,094 participants were included (Figure 2, upper part) to study the co‐variation of the studied variables without a priori assumptions. Diabetes and hypertension are well‐known risk factors for cardiac events, and their combination results in an additional risk increase for cardiovascular events including SCD. In addition, both conditions are determinants for the QRS‐T angles (Delhey, Jin, Thapa, Delongchamp, & Faramawi, 2020; Whang et al., 2012). A post hoc study was therefore conducted to assess the correlation between inflammatory markers and wide QRS‐T angles independent of diabetes and hypertension (Figure 2, lower part).

### 2.2 Design of the post hoc study

Among all participants eligible for matching, 311 had hypertension, 33 had diabetes and 53 had both diagnoses. Among the remaining participants, 320 "healthy" participants without acute or chronic disease, chronic medication, or abnormal laboratory tests requiring medical attention were identified. 52 participants eligible for matching had wide QRS‐T angles and constituted the study group with wide QRS‐T angles ("QRS‐T_W_"). Two age‐ and sex‐matched groups with the same number of participants were then established. One of the two groups was further matched with regard to the presence of hypertension, diabetes, both, or neither ("HT/DM"). The second group was not matched for disease and therefore constituted a healthy control group ("Healthy"). For a detailed comparison of the three study groups, see online Table S1.

Participants who were not eligible for matching due to various diseases were excluded (*n* = 377, Figure 1). These included for example participants with cancer and rheumatic diseases, and also participants with dyspnea or exertional chest discomfort without defined etiologies. In the matched group design, 30 participants with wide QRS‐T angles were thus excluded as proper matching was not possible. They are, however, included in the comparisons in Table 1.

**TABLE 1:** Characteristics of the population sample and comparisons between participants with and without wide QRS‐T angles

Categories	All participants (*n* = 1,094)	Wide QRS‐T angles (*n* = 82)	Not wide QRS‐T angles (*n* = 1,012)	*p*‐value^a^
Women/men (*n*)	550/544	28/54	522/490	<.01
**Variable**	**Median (Q1–Q3)**	**Median (Q1–Q3)**	**Median (Q1–Q3)**	
Age (years)	57.6 (53.8–61.7)	58.6 (55.6–62.5)	57.5 (53.7–61.6)	NS
Weight (kg)	80.0 (69.0–89.9)	87.1 (74.7–96.5)	79.4 (68.8–89.2)	<.001
Height (m)	1.71 (1.64–1.79)	1.72 (1.67–1.80)	1.71 (1.64–1.78)	NS
BMI (kg/m^2^)	26.6 (24.4–29.4)	28.5 (25.9–32.0)	26.5 (24.3–29.4)	<.001
**Disease history**	***n* (%)**	***n* (%)**	***n* (%)**	
Myocardial infarction	12 (1.1)	1 (1.2)	11 (1.1)	NS
Coronary revascularization	19 (1.7)	2 (2.4)	17 (1.7)	NS
Heart failure	10 (0.9)	2 (2.4)	8 (0.8)	NS
Valve disease	3 (0.3)	1 (1.2)	2 (0.2)	NS
Stroke	11 (1.0)	1 (1.2)	10 (0.1)	NS
Atrial fibrillation^b^	30 (2.7)	4 (4.9)	26 (2.6)	NS
Hypertension	364 (33)	36 (44)	328 (32)	<.05
Diabetes	86 (7.9)	17 (21)	69 (6.8)	<.001
Cancer	80 (7.3)	5 (6.1)	75 (7.4)	NS
Rheumatic disease	74 (6.8)	8 (10)	66 (6.5)	NS
Apparently healthy	320 (17)	9 (11)	311 (31)	<.001
Prescribed medication	489 (45)	50 (61)	439 (43)	<.01
**Smoking habits**	***n* (%)**	***n* (%)**	***n* (%)**	
Never smoked	472 (43)	30 (37)	442 (44)	NS
Active smoker	161 (15)	14 (17)	147 (15)	NS
Occasional smoker	36 (3.3)	2 (2.4)	34 (3.4)	NS
Ex‐smoker	421 (38)	35 (43)	386 (38)	NS
**Blood analyses**	**Median (Q1–Q3)**	**Median (Q1–Q3)**	**Median (Q1–Q3)**	
Cholesterol (total) (mmol/L)	5.7 (5.0–6.5)	5.6 (4.7–6.3)	5.7 (5.1–6.5)	NS
LDL (mmol/L)	3.8 (3.1–4.4)	3.6 (3.0–4.3)	3.8 (3.1–4.4)	NS
HDL (mmol/L)	1.6 (1.3–2.0)	1.6 (1.2–1.8)	1.6 (1.3–2.0)	NS
Triglycerides (mmol/L)	1.1 (0.8–1.6)	1.2 (0.9–1.7)	1.1 (0.8–1.6)	NS
Apo B/Apo A1 ratio (unitless)	0.66 (0.54–0.81)	0.65 (0.55–0.83)	0.66 (0.54–0.81)	NS
Glucose (mmol/L)	5.6 (5.2–6.1)	5.8 (5.4–6.5)	5.6 (5.2–6.0)	<.01
HbA1c (mmol/mol)	35 (33–38)	37 (34–40)	35 (33–38)	<.01
Hemoglobin (g/L)	140 (132–149)	144 (134–150)	140 (132–148)	<.05
ALT (µkat/L)	0.43 (0.34–0.57)	0.44 (0.34–0.59)	0.43 (0.34–0.57)	NS
hsCRP (mg/L)	1.3 (0.6–2.8)	1.6 (0.8–3.1)	1.3 (0.6–2.7)	NS
Creatinine (µmol/L)	78 (69–88)	81 (72–89)	77 (68–87)	NS

^a^ Mann‐Whitney, Fisher's exact test, or chi‐square test.

^b^ Including 4 newly diagnosed cases among 9 with this arrhythmia during the VCG recording.

### 2.3 VCG recording and analysis

Vectorcardiography based on orthogonal (XYZ) recordings according to Frank was used, which is regarded as the “gold standard” for assessing the spatial QRS‐T angles (Frank, 1956; Schreurs et al., 2010). The VCG recording methodology and analysis were performed following the same principles used by our group as described previously, using the same terminology and definitions of parameters (Lingman et al., 2016). A ≥5 min recording was planned after 5 min at supine rest with closed eyes and no conversation to allow heart rate stabilization and heart rate adaption of ventricular repolarization. The CoroNet II system (Ortivus AB) was used and 8 electrodes positioned: 5 around the chest at the level of the insertion of the 5th rib on the sternum, one in the neck, and one each on the left and right hip. Signals from the 3 orthogonal XYZ leads were sampled at 500 Hz with an amplifier bandwidth of 0.03–170 Hz. After the resting period of ≥5 min, the most stable sampling period of 10 s was selected and used for analysis applying a customized algorithm. The CoroNet software automatically selected annotation points for P‐wave start, QRS onset, peak and offset, and T‐wave peak and end. A signal‐averaged QRST complex as well as QRS‐ and T‐vector loops were based on all cardiac cycles of dominant shape and with good signal quality during this 10‐s period. Heart rate in beats per minute (bpm) was defined from this 10‐s period.

### 2.4 QRS‐T angles

We computed two measures of the relation between the dominant depolarization (QRS) and repolarization (T) forces in space (3‐D): (a) the spatial peak QRS‐T angle between the maximum QRS‐ and T‐vectors inscribed in the QRS‐ and T‐vector loops (also referred to as the "QRS‐T angle"); and (b) the spatial mean QRS‐T angle between the QRS area and the T area vectors (also referred to as the "QRS‐T area angle"; Lingman et al., 2016; Oehler et al., 2014). Wide angles were defined as the presence of a peak QRS‐T angle >123° and/or a mean QRS‐T angle >112°. These cutoff values were derived from an earlier study using similar vectorcardiographic methodology that showed the additional prognostic value of these measures (beyond age, sex, diabetes, stroke, left ventricular ejection fraction, estimated glomerular filtration rate, as well as heart rate, systolic blood pressure <100 mmHg, and Killip class >1 on hospital admission) with regard to both SCD and all cardiac deaths after acute coronary syndromes applying c‐statistics and reclassification analysis (Lingman et al., 2016).

### 2.5 Laboratory analyses

Venous blood samples were obtained from all study subjects using vacutainer tubes. Complete blood counts, including white blood cell count (WBC), neutrophils, lymphocytes, monocytes, and eosinophils were measured with the auto‐hematology analyser ADVIA^®^ 2120i System (Siemens Healthcare GmbH, Erlangen, Germany). HemoglobinA1c (HbA1c) were analyzed by the approved reference method using high pressure liquid chromatography system with UV detection (Jeppsson et al., 2002).

Serum levels of alanine aminotransferase (ALT), cholesterol, triglycerides, high‐density lipoprotein cholesterol (HDL), low‐density lipoprotein cholesterol (LDL), apolipoprotein A (apoA), apolipoprotein B (apoB), glucose, creatinine, cystatin C, C‐reactive protein (CRP), cardiac troponin t (cTnT), and N‐terminal pro b‐type natriuretic peptide (NT‐pro‐BNP) were analyzed using standard laboratory techniques and automated Cobas instruments (Roche Molecular Diagnostics). All analyses were performed at the Department of Clinical Chemistry, Sahlgrenska University Hospital. Estimates of glomerular filtration rate were calculated based on creatinine and cystatin C as previously described (Grubb et al., 2014; Nyman et al., 2014). Means of the two estimates were calculated and used for further analyses and presentation.

Levels of pro‐inflammatory cytokines were measured in serum samples using the ultrasensitive multiplex electrochemiluminescence immunoassay (ELISA; Meso Scale Diagnostics). The human V‐PLEX Pro‐inflammatory Panel 1 Human Kit (#K15049) for the precise quantitative determination of interferon gamma (IFN‐γ), interleukin (IL)‐1β, IL‐2, IL‐4, IL‐6, IL‐8, IL‐10, IL‐12p70, IL‐13, and tumor necrosis factor‐alpha (TNF‐α) were applied. Intensity of the emitted light was measured according to the manufacturer's instructions on an MSD QuickPlex SQ120 plate reader (Meso Scale Diagnostics).

### 2.6 Coronary calcium score

Imaging for coronary artery calcification score (CACS) was performed using a dedicated dual‐source CT scanner equipped with a Stellar Detector (Siemens, Somatom Definition Flash, Siemens Medical Solution; Gummesson et al., 2018).The calcium content in each coronary artery was measured and summed to produce a total CACS as described previously (Bergstrom et al., 2015).

### 2.7 Statistics

Nondetectable levels of analytes and coronary calcium scores were assigned numerical values corresponding to half of the lowest level detected for the respective variables. All data were log transformed prior to further statistical analysis. The study participants were randomly divided into a training set (*n* = 539) and a validation set (*n* = 548). Principal component analysis (PCA) was performed to determine the correlation structure of 31 variables reflecting impaired glucose tolerance and diabetes, hypertension, cardiovascular disease, age, inflammation, heart disease, and wide QRS‐T angles. Orthogonal Projections to Latent Structures (OPLS) models for the prediction of QRS‐T angles were also determined to calculate the regression coefficients of the respective X‐variables. Outliers were determined as individuals with scores >Hotellings T2 99% level and/or a DmodX level >2 times 95% Dcrit level. A few outliers were also excluded based on t1/u1 plots for the OPLS models, as these individuals clearly deviated from the majority of observations. The PCA and OPLS models were firstly calculated based on the training data set, secondly based on the validation data set to determine the reproducibility of the models. PCA and OPLS models were calculated with as well as without outliers.

Correlations between wide QRS‐T angles and markers of inflammation, cardiac injury, and metabolism were corrected for hypertension, diabetes, age, and sex through the previously described matching between study groups. To minimize the number of comparisons, Student's *t* tests were only calculated for the study participants with wide QRS‐T angles in comparison with healthy controls and participants matched for hypertension and diabetes, respectively. Mann‐Whitney tests were calculated when the conditions for Student's *t* tests were not met.

All statistical analyses were calculated using SPSS v. 25 (IBM) or Simca v. 15 (Sartorius Stedim Data Analytics AB). *p* values <.05 were considered significant. Data are presented as median and 95% confidence interval unless otherwise stated. Graphs were constructed using GraphPad Prism v. 8.

## 3 RESULTS

### 3.1 Study participants

Table 1 shows characteristics of all study participants combined and divided by subgroups with and without wide QRS‐T angles. Diabetes and hypertension were significantly more common (and, accordingly, BMI, glucose, and HbA1c were higher) among those with wide QRS‐T angles. For the majority of the 82 participants with wide QRS‐T angles, the cause of the electrophysiological abnormalities was not known. However, 6 had left bundle branch block, 2 had right bundle branch block with either left anterior or posterior fascicular block, and 3 had known heart disease without bundle branch block.

### 3.2 Multivariate data analysis

**FIGURE 3:**
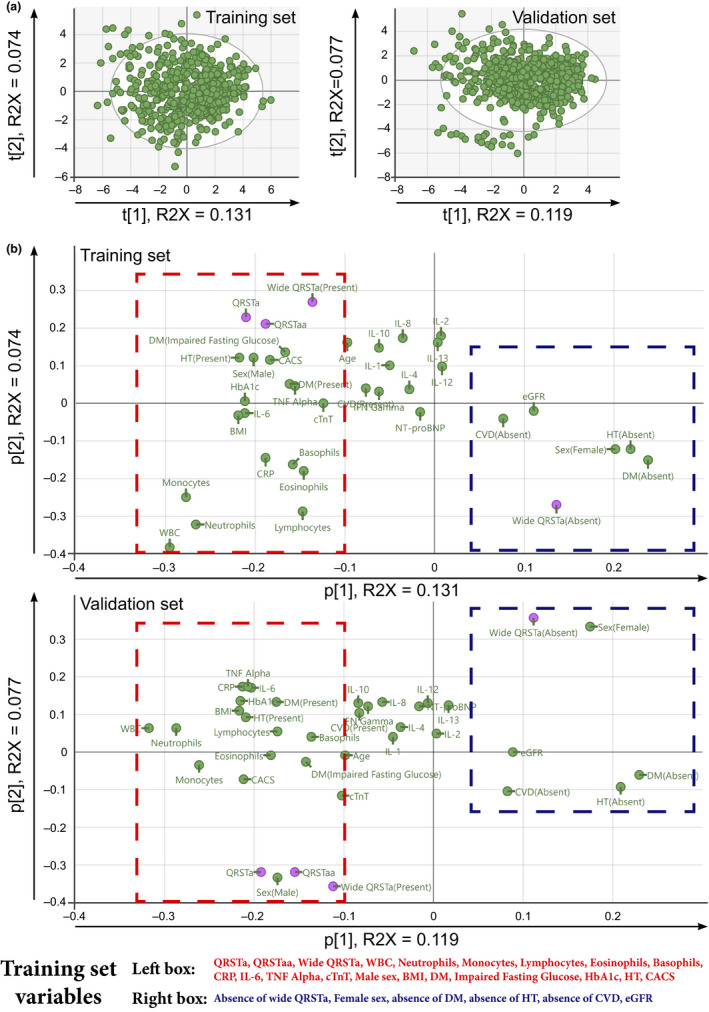
Correlations between the QRS‐T angle, age, sex, hypertension, diabetes, and markers of inflammation, heart disease, and metabolism. Principal component analyses (PCA) were calculated based on two groups of randomly selected study participants ("training" and "validation set," 30 and 19 outliers excluded, respectively). (a) Score plots with ellipses indicating the boundaries of Hotellings T2 95% level. (b) Loading plots. QRS‐T angle, area angle, and pathologically wide QRS‐T angle (purple markers) are clustered, indicating positive correlation between the variables. The three variables also correlate positively with presence of diabetes, impaired fasting glucose, hypertension, male sex, age, BMI, cTnT, HbA1c, CACS as well as CRP, IL‐6, TNF‐α, and the cell count of the respective types of leukocytes analyzed. Rectangles of equal size have been outlined to contain clustering variables, based on the first principal component of each model. Below the score plots, variables within the outlined rectangles of the training set score plot have been noted. All variables fell within the same rectangle for the validation set and have been highlighted in bold, color coded font. CACS, Coronary Calcium Score; CRP, C‐reactive protein; cTnT, cardiac troponin T; CVD, cardiovascular disease; DM, diabetes mellitus; eGFR, estimated glomerular filtration rate; HT, hypertension; QRSTa, peak QRS‐T angle; QRSTaa, mean QRS‐T angle; WBC, white blood cell count

Principal component analysis was calculated based on 539 randomly selected study participants ("training set," 30 outliers excluded prior to analysis, Figure 3a). PCA was chosen to determine the correlation between the analyzed variables without a priori assumptions, while at the same time allowing for even highly co‐varying variables to be included. This resulted in a model where two principal components (PC) contributed significantly to the model. The continuous variables QRS‐T and QRS‐T area angle, as well as the dichotomous presence of a wide QRS‐T angle, were strongly, positively correlated as determined by their similar loadings (Figure 3b). These variables also correlated positively with the presence of diabetes, impaired fasting glucose, HbA1c, hypertension, male sex, age, BMI, cardiac troponin T (cTnT), and coronary calcium score (CACS) as well as with CRP and the cell count of several different leukocyte types. The cytokines IL‐6 and TNF‐α also correlated positively with the QRS‐T angles.

Similar models and correlation patterns were observed when the first principal component of the PCA was validated on the remaining 548 study participants ("validation set," 19 outliers excluded prior to analysis). The second principal component of the PCA model was not fully reproducible, which corresponded to a lower explanatory value of the component in both calculated models. Diabetes, impaired fasting glucose, HbA1c, hypertension, male sex, BMI, cTnT, CACS, WBC count, the number of all leukocyte types analyzed, and the levels of CRP, IL‐6, and TNF‐α were reproducibly, positively correlated with the QRS‐T angles. In contrast, female sex, estimated glomerular filtration rate (eGFR), and the absence of diabetes, hypertension, and cardiovascular disease correlated negatively with the QRS‐T angles. PCA models calculated without the omission of outliers resulted in similar models (Figure S1).

**FIGURE 4:**
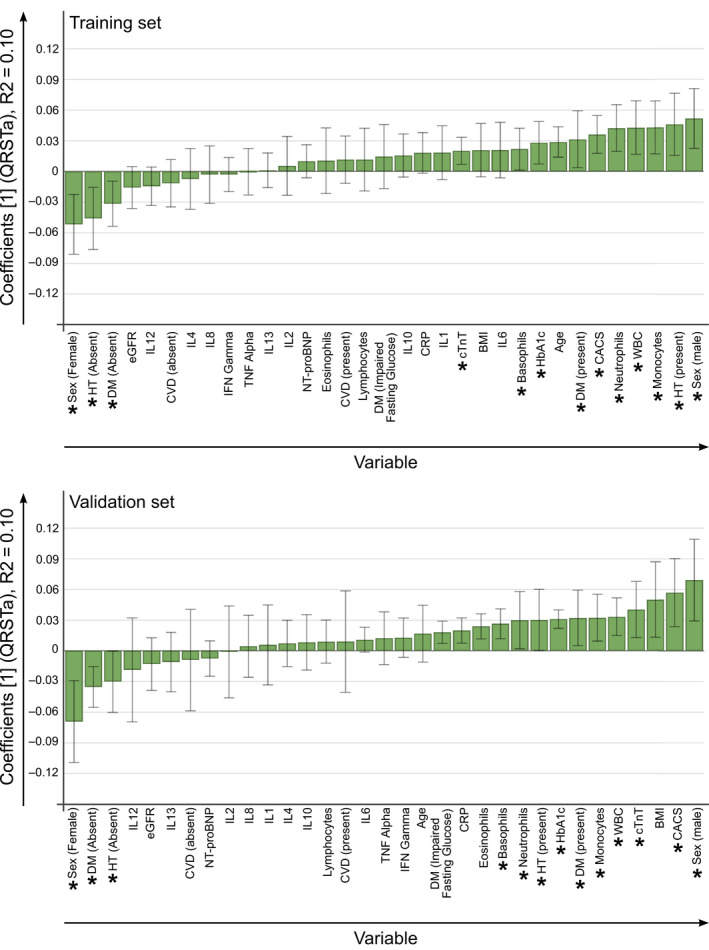
Prediction of the QRS‐T angle width based on age, sex, hypertension, diabetes, and markers of inflammation, heart disease, and metabolism. OPLS models were calculated based on two groups of randomly selected study participants ("training" and "validation set," 3 and 10 outliers excluded, respectively). Regression coefficients and 95% confidence intervals are shown. Significant predicting variables, which were reproduced in the model based on the validation data set, have been marked with "*". Male sex, diabetes, HbA1c, hypertension, CACS, cTnT as well as WBC and the number of neutrophils, monocytes, and basophils were significant predicting factors for larger QRS‐T angles for both model sets. Female sex and absence of diabetes or hypertension on the other hand constituted significant predicting factors for smaller QRS‐T angles. CACS, Coronary Calcium Score; CRP, C‐reactive protein; cTnT, cardiac troponin T; CVD, cardiovascular disease; DM, diabetes mellitus; eGFR, estimated glomerular filtration rate; HT, hypertension; QRSTa, QRS‐T angle; WBC, white blood cell count

To determine regressions coefficients for the analyzed variables, OPLS models predicting QRS‐T angle width were calculated for the training and validation sets (Figure 4; 3, and 10 outliers excluded, respectively). Male sex, diabetes, hypertension, CACS, HbA1c, cTnT, and cell count of different leukocytes were significant predictors for wide QRS‐T angles. In contrast, female sex, and absence of diabetes or hypertension constituted significant predictors for narrower QRS‐T angles. The patterns of regression coefficients were similar between the training and validation sets. Notably, WBC and the number of neutrophils, monocytes, and basophils constituted significant variables for the prediction of QRS‐T angle width for both models. OPLS models calculated without the omission of outliers resulted in similar models (Figure S2).

### 3.3 Post hoc study based on matched study groups

A post hoc study was performed to determine whether the observed positive correlations between markers of inflammation and QRS‐T angle width were independent of hypertension, diabetes, age, and sex. Three subgroups were formed—one with wide QRS‐T angles, one without wide QRS‐T angles matched for age, sex, diabetes, and hypertension, and one healthy control group matched for age and sex (Table S1).

**FIGURE 5:**
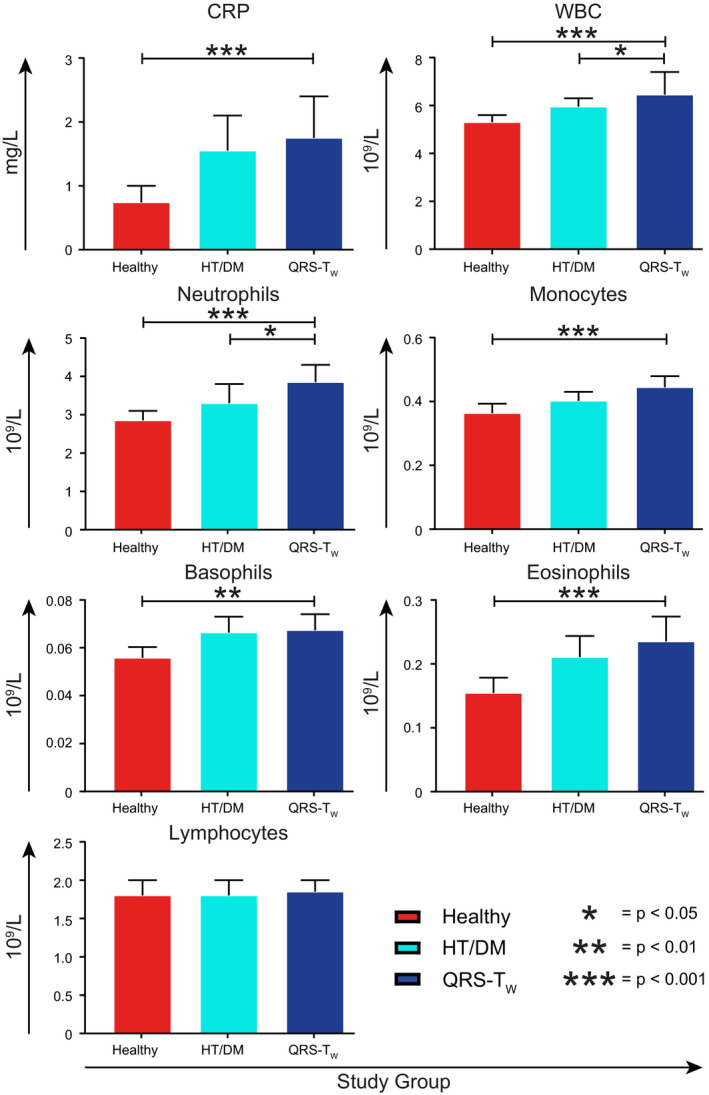
Concentrations of established inflammatory markers. Participants with wide QRS‐T angles (QRS‐T_W_) were compared with healthy controls (Healthy) and individuals matched for hypertension and diabetes (HT/DM) using Student's *t* tests. Data are presented as median and 95% confidence interval (CI), except for monocytes, basophils, and eosinophils where mean and 95% CI is used. For these three variables, the mean was a more exact measure due to the relatively low measurement accuracy and high number of nondetectable values. *p < .05, **p < .01, ***p < .001. CRP, C‐reactive protein; WBC, white blood cell count

Study participants with wide QRS‐T angles had significantly higher levels of WBC and number of neutrophils in comparison with both control groups, as well as significantly higher levels of CRP, monocytes, basophils, and eosinophils in comparison with healthy controls (Figure 5).

**FIGURE 6:**
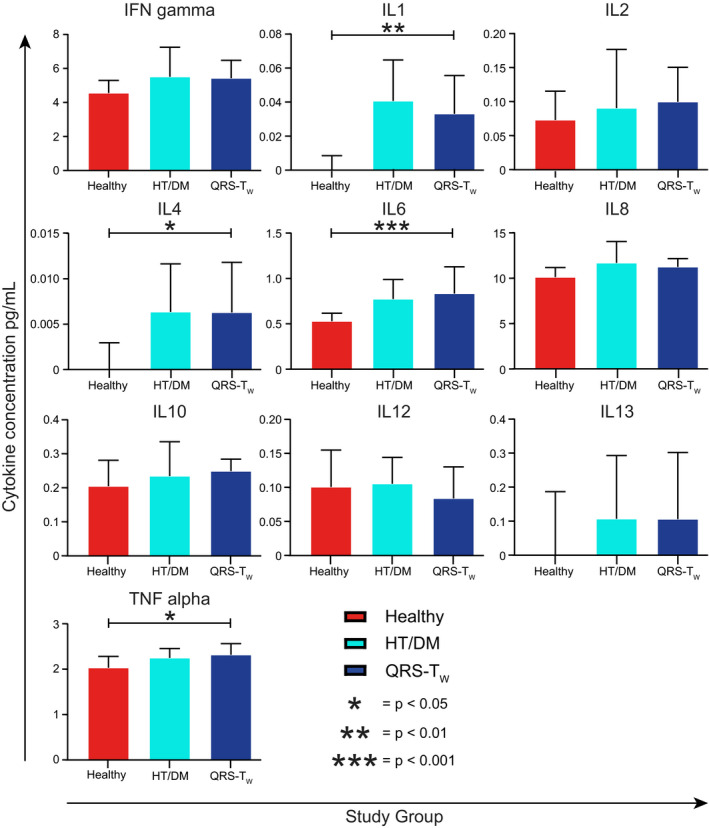
Serum cytokine levels in study participants. Participants with wide QRS‐T angles (QRS‐T_W_) were compared with healthy controls (Healthy) and individuals matched for hypertension and diabetes (HT/DM) using Student's *t* tests. Many participants in the healthy control group had nondetectable IL‐1, IL‐4, and IL‐6 levels, resulting in low median values. Data are presented as median and 95% confidence interval. *p < .05, **p < .01, ***p < .001

**FIGURE 7:**
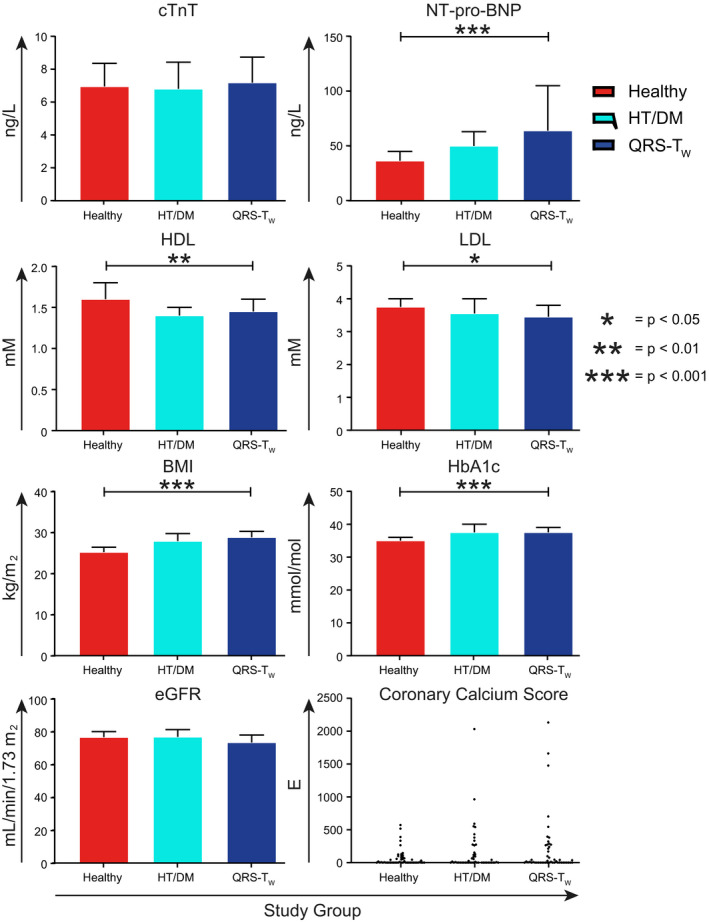
Markers of cardiac wall stress/injury, coronary atherosclerosis, and the metabolic syndrome in study participants. Participants with wide QRS‐T angles (QRS‐T_W_) were compared with healthy controls (Healthy) and individuals matched for hypertension and diabetes (HT/DM) using Student's *t* tests. Data are presented as median and 95% confidence interval except for the coronary calcium score. *p < .05, **p < .01, ***p < .001. cTnT, cardiac troponin T, eGFR, estimated glomerular filtration rate

Participants with wide QRS‐T angles also had significantly higher concentrations of the inflammatory cytokines IL‐1, IL‐4, IL‐6, and TNF‐α in comparison with healthy controls (Figure 6). There were however no significant differences in cytokine concentrations between participants with wide QRS‐T angles and those matched for hypertension and diabetes. Finally, study participants with wide QRS‐T angles had significantly higher concentrations of NT‐pro‐BNP and HbA1c as well as BMI in comparison with healthy controls (Figure 7). HDL and LDL concentrations on the other hand were significantly lower in study participants with wide QRS‐T angles. There were no significant differences between participants with wide QRS‐T angles and those matched for hypertension and diabetes. Coronary calcium scores did not differ significantly between groups.

## 4 DISCUSSION

The main finding of this study was that wide QRS‐T angles were associated with markers of inflammation, cardiac wall stress/injury, the metabolic syndrome, and male sex. Inflammatory markers were positively correlated with increased QRS‐T angle width, independent of age, sex, hypertension, and diabetes. These results corroborate an association between inflammatory activation and abnormal electrophysiology, and may have implications for SCD prediction.

Inflammatory mechanisms, for example, through activation of T cells and macrophages, have been implicated in the development and progress of ischemic heart disease as well as heart failure (Bansal et al., 2017; Barisione et al., 2010; Okamoto et al., 2014; Tsujioka et al., 2009; Weirather et al., 2014). Inflammatory activation may also have a direct impact on SCD risk. Cytokines such as IL‐1 and TNF‐α may for example have arrhythmogenic effects by altering the electrophysiological function of cardiomyocytes (Baum et al., 2012; Fernandez‐Velasco et al., 2007; Petkova‐Kirova et al., 2006). Radical oxygen species (ROS) produced during inflammatory reactions may also be a link between inflammation, abnormal and wide QRS‐T angles, and SCD. ROS may activate CaMKII and result in alterations in cardiac electrophysiological function and structure (Liu et al., 2019; Singh & Anderson, 2011). IL‐6 is another example of an important inflammatory mediator, which may induce maladaptive hypertrophy as well as a decreased contractile function of cardiomyocytes (Fontes, Rose, & Cihakova, 2015). Elevated IL‐6 levels in peripheral blood have predicted SCD in several population‐based cohorts (Empana et al., 2010; Hussein et al., 2013; Parekh et al., 2008). The association between increased IL‐6 levels and wide QRS‐T angles in the present study is therefore in line with previous observations. WBC has in one previous study been shown to predict SCD (Kucharska‐Newton et al., 2009). Our findings corroborate the association between WBC and an elevated risk for SCD. While the number of several leukocyte types was higher in participants with wide QRS‐T angles as compared to healthy controls, only the number of neutrophils was significantly higher when compared to participants matched for hypertension and diabetes. Our findings therefore suggest a previously unknown role for neutrophils in SCD prediction. Production of ROS constitutes one possible link between neutrophils, wide QRS‐T angles, and SCD and warrants further study.

Cytokines were not significantly elevated in the group with wide QRS‐T angles when compared to participants matched for hypertension and diabetes. While cytokines are key signals in the localized milieu of an inflamed tissue, the local concentrations may not be sufficiently elevated to affect the concentrations in the peripheral blood. The relatively short half‐life of cytokines (Adabi, Saebi, Moradi Hasan‐Abad, Teimoori‐Toolabi, & Kardar, 2017) in comparison with for example CRP (Kuribayashi et al., 2015) may also increase the variability of measurements, thereby limiting the potential to detect elevated cytokine levels. While cytokines may have an arrhythmogenic role in the stressed/injured myocardium, it might therefore be preferable to utilize for example WBC or the number of neutrophils in SCD risk prediction algorithms.

The potential role for CRP in predicting SCD has previously been studied—with conflicting results (Albert, Ma, Rifai, Stampfer, & Ridker, 2002; Empana et al., 2010; Korngold et al., 2009; Parekh et al., 2008). In one study, patients suffering from the metabolic syndrome demonstrated wider QRS‐T angles, and a correlation between CRP levels and QRS‐T angles was observed (Voulgari et al., 2010). While CRP was associated with wider QRS‐T angles in our study, there was no significant difference in comparison with the matched control group—indicating that the difference may at least in part be explained by the presence of hypertension and/or diabetes.

No significant differences were observed for markers of cardiac wall stress/injury, coronary atherosclerosis, and components of the metabolic syndrome when study participants with wide QRS‐T angles were compared to controls matched for diabetes and hypertension. This supports the validity of the matched design, and in turn the positive independent correlation between inflammatory markers and QRS‐T angle width. The elevated NT‐pro‐BNP concentrations observed in the group with wide QRS‐T angles as compared to the healthy control group are signs of cardiac wall stress and/or injury. BMI and HbA1c were higher for the group with wide QRS‐T angles as compared to the healthy controls, probably due to the co‐variation between obesity, diabetes, and hypertension. The lower LDL levels in the group with wide QRS‐T angles in comparison with healthy controls may reflect that the former group received treatment as part of the overall attempts to reduce the effects of hypertension and diabetes on cardiovascular events. The significantly lower HDL levels observed indeed support the assumption that the participants with wide QRS‐T angles demonstrated some degree of dyslipidemia.

### 4.1 Study limitations

Principal component analysis and OPLS models may be used to visualize complex correlation structures between variables, as well as to predict one or several outcome variables. By using PCA, which is an unsupervised model without a priori assumptions, three measures of QRS‐T angle width (two continuous and one categorical) were shown to correlate with risk factors of heart disease and SCD. OPLS models developed to predict QRS‐T angle width demonstrated similar results, as shown by the regression coefficients for the predictor variables. To verify our findings, all study participants had randomly been assigned to either a "training set" or a "validation set" before development of the PCA/OPLS models. The similar results obtained for both data sets suggest that the observed correlations are not due to random variation. Notably, the WBC and the numbers of neutrophils were associated with increased QRS‐T angle width in all models and data sets. WBC, and the numbers of neutrophils were also significantly increased in the group with wide QRS‐T angles as compared to matched controls. These observations indicate that inflammatory activation is not only an effect of hypertension, diabetes, the metabolic syndrome, male sex, or old age, but is specifically associated with wide QRS‐T angles. The results from the PCA/OPLS models based on the entire cohort were thus corroborated.

Several in vitro studies point toward a possible causal relationship between cardiac inflammation, ischemic heart disease, and arrhythmogenesis—as discussed previously. As the current study is based on cross‐sectional observational data, it is not possible to prove any causal relationships between inflammatory activation, wide QRS‐T angles, and SCD. The role of cardiac inflammation in arrhythmogenesis and SCD therefore requires further study in humans.

Previous studies on the relationship between abnormal QRS‐T angles and subsequent SCD have analyzed the QRS‐T angle as a categorical variable. As statistical power increases when variables are treated as continuous, rather than categorical, OPLS models were calculated to predict the continuous QRS‐T angle width. All other statistical models, however, included the categorical, binary, wide QRS‐T angle according to specified cutoff values. The prognostic impact of wide QRS‐T angles has not yet been tested within the SCAPIS study. The reviews by Oehler et al. and Voulgari et al., however, present compelling evidence of the predictive value of abnormal QRS‐T angles in various cohorts despite different electrocardiographic methods. Our cutoff values were similar (Oehler et al., 2014; Voulgari, Pagoni, Tesfaye, & Tentolouris, 2013). Furthermore, the cutoff values used in this study have previously been shown to contribute additional prognostic value on top of 9 standard variables using c‐statistic and reclassification analysis after acute coronary syndrome (Lingman et al., 2016).

## 5 CONCLUSIONS

Markers of inflammation and cardiac wall stress and injury were higher in individuals with wide QRS‐T angles. Our results support an association between inflammatory activation, altered electrophysiology, and increased SCD risk. The role of inflammation and wide QRS‐T angles in the pathophysiology and prediction of SCD warrants further study.

## CONFLICT OF INTEREST

None.

## ETHICAL APPROVAL

Ethical approval was obtained from the local Ethical Review Board at the University of Gothenburg (December 8, 2016, #1009‐16). The study was carried out in accordance with the Helsinki Declaration, as revised 2013.

## Supporting information

Fig S1

Fig S2

Table S1
