# Supplementary material for: Wide QRS‐T angles are associated with markers of increased inflammatory activity independently of hypertension and diabetes
Source: Ann Noninvasive Electrocardiol. 2020 Jul 8;25(6):e12781. doi: 10.1111/anec.12781 (PMC7679831; doi:10.1111/anec.12781)
Supplement: Supplementary file 1 — Fig S1 [file ANEC-25-e12781-s001.pdf]

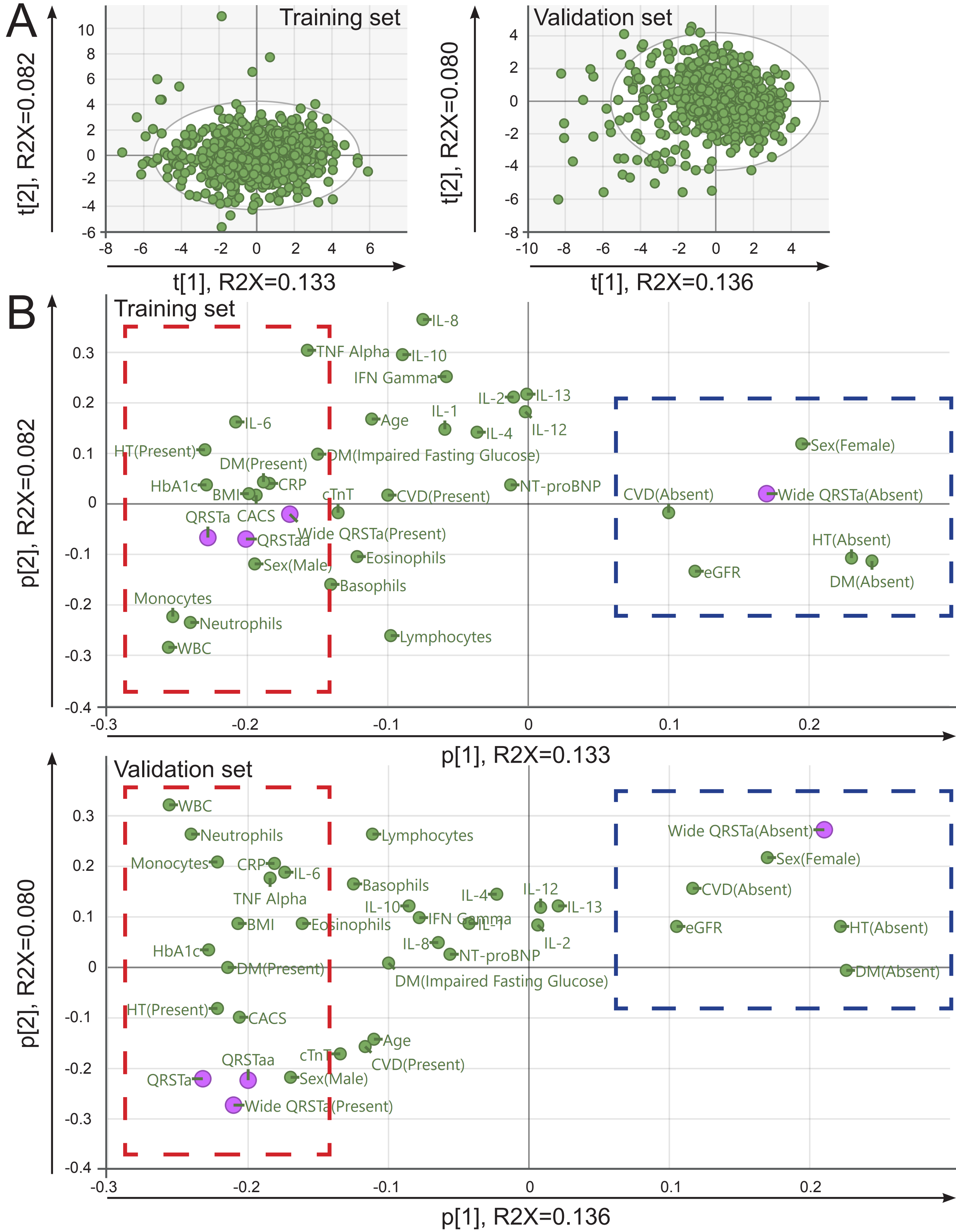

Training set variables

Left box: **QRSTa, QRSTaa, Wide QRSTa, WBC, Neutrophils, Monocytes, CRP, IL-6, TNF Alpha, Male sex, BMI, DM, HbA1c, HT, CACS**, *Impaired Fasting Glucose.*

Right box: **Absence of wide QRSTa, Female sex, absence of DM, absence of HT, absence of CVD, eGFR**
