# Supplementary figures and images for: Wide QRS‐T angles are associated with markers of increased inflammatory activity independently of hypertension and diabetes
Source: Ann Noninvasive Electrocardiol. 2020 Jul 8;25(6):e12781. doi: 10.1111/anec.12781 (PMC7679831; doi:10.1111/anec.12781)

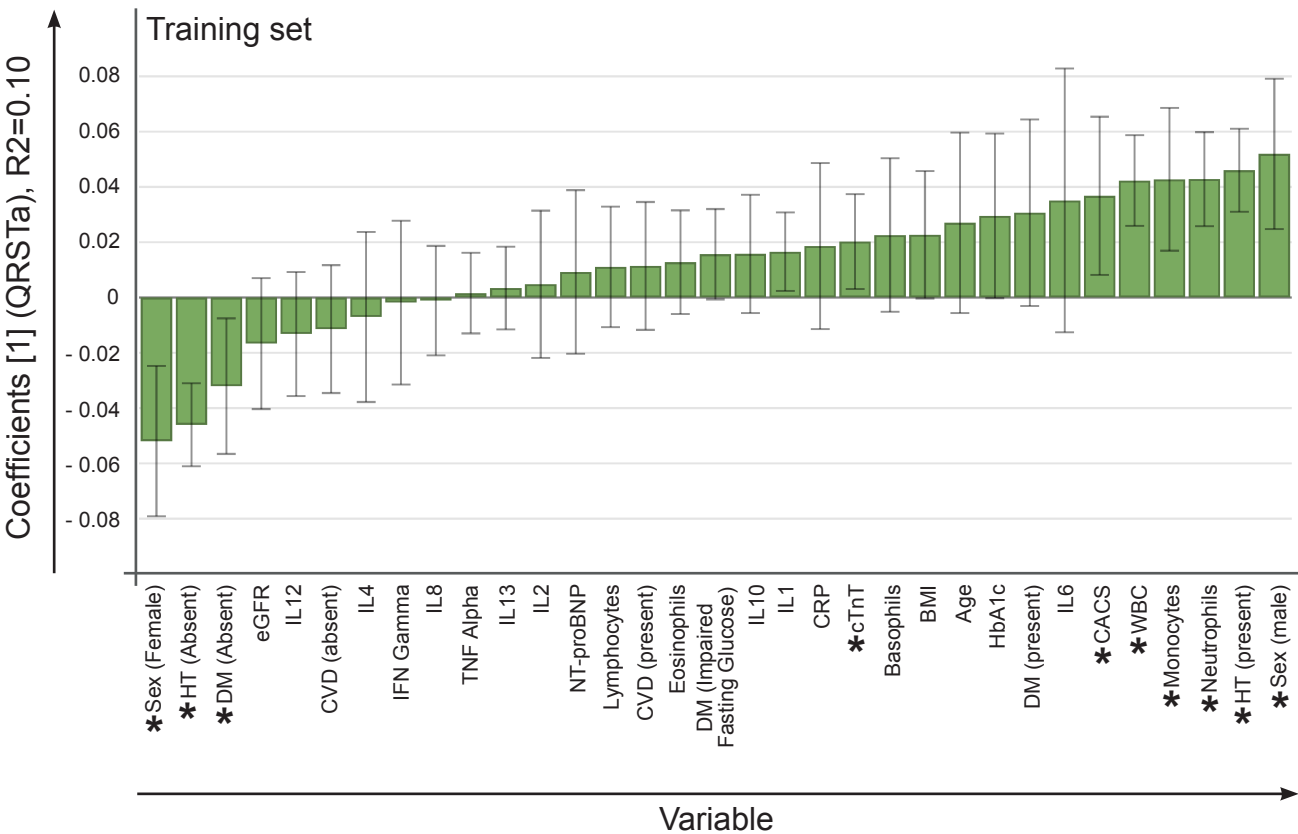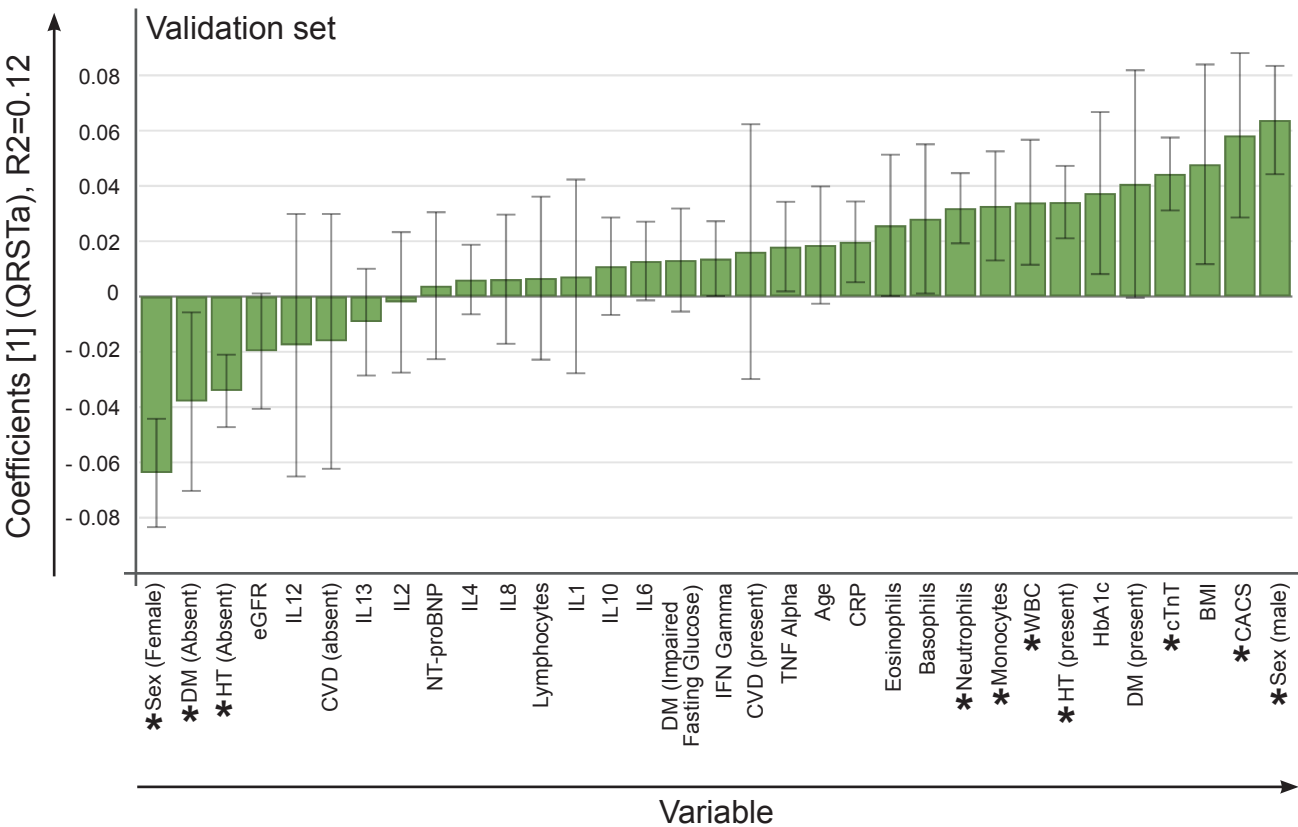

Supplement: Supplementary file 2 — Fig S2 [file ANEC-25-e12781-s002.pdf]
