# Supplementary material for: Wide QRS‐T angles are associated with markers of increased inflammatory activity independently of hypertension and diabetes
Source: Ann Noninvasive Electrocardiol. 2020 Jul 8;25(6):e12781. doi: 10.1111/anec.12781 (PMC7679831; doi:10.1111/anec.12781)
Supplement: Supplementary file 3 — Table S1 [file ANEC-25-e12781-s003.docx]

| **Supplemental table 1.** Characteristics of the three sex- and age-matched subgroups of 52 participants each. Data presented as n (%) for categories, otherwise median and interquartile range (Q1; Q3). | | | |
| --- | --- | --- | --- |
| **Group category** | **Healthy** | **Matched for HT/DM** | **Wide QRS-T angles** |
| Females/Males | 14/38 | 14/38 | 14/38 |
| Age [yrs] | 59.6 (55.0; 62.8) | 59.4 (55.0; 62.8) | 59.2 (55.0; 62.7) |
| Hypertension (HT) | 0 | 26 (50) | 26 (50) |
| Diabetes Mellitus (DM) | 0 | 7 (13) | 7 (13) |
| HT + DM | 0 | 10 (19) | 10 (19) |
| Healthy | 52 (100) | 9 (17) | 9 (17) |
| BMI [kg/m^2^] >27 <30  ≥30 | 5 (10)  8 (15) | 9 (17)  19 (37) | 11 (21)  23 (44) |
| BMI [kg/m^2^] | 25.2 (22.8; 27.6) | 27.9 (24.9; 31.6) | 28.8 (26.2; 33.0) |
| Cholesterol >5.5 and/or  LDL>3.5 [mmol/L] | 32 (62) | 26 (50) | 25 (48) |
| Total cholesterol [mmol/L] | 5.7 (4.9; 6.4) | 5.4 (4.7; 6.3) | 5.3 (4.4; 6.0) |
| LDL [mmol/L] | 3.8 (3.1; 4.4) | 3.6 (3.0;4.2) | 3.5 (2.7; 4.1) |
| HDL [mmol/L] | 1.6 (1.4; 2.1) | 1.4 (1.2; 1.7) | 1.5 (1.2; 1.7) |
| Triglycerides [mmol/L] | 1.0 (0.7; 1.4) | 1.2 (0.9; 2.0) | 1.3 (0.8; 1.7) |
| Apo B/Apo A1 [unitless] | 0.63 (0.53; 0.80) | 0.67 (0.61; 0.84) | 0.67 (0.56; 0.82) |
| Glucose ≥7 [mmol L^/^] and/or  HbA1c >48 [mmol/mol] | 0 | 21 (40) | 15 (29) |
| Glucose [mmol/L] | 5.6 (5.3; 6.0) | 6.3 (5.6; 7.3) | 5.9 (5.5; 7.0) |
| HbA1c [mmol/mol] | 35 (33; 37) | 38 (36; 46) | 38 (35; 46) |
| ALT [µkat/L] | 0.40 (0.31; 0.53) | 0.50 (0.40; 0.70) | 0.44 (0.33; 0.63) |
| CRP > 5 [mg/L] | 0 | 9 (17) | 6 (12) |
| CRP [mg/L] | 0.7 (0.4; 1.3) | 1.6 (0.9; 3.5) | 1.8 (0.6; 3.1) |
| Creatinine [μmol/L] | 81 (72; 90) | 81 (71; 90) | 83 (72; 91) |
| Peak QRS-T angle [°] | 24 (17; 35) | 31 (22; 47) | 131 (115; 155) |
| Mean QRS-T angle [°] | 42 (30; 61) | 49 (34; 75) | 134 (115; 148) |
